# A population-based comparison of patients with metastatic esophagogastric carcinoma between Japan and the Netherlands

**DOI:** 10.1007/s00432-023-05111-4

**Published:** 2023-07-24

**Authors:** Marieke Pape, Pauline A. J. Vissers, Ken Kato, Nadia Haj Mohammad, Bastiaan Klarenbeek, Hanneke W. M. van Laarhoven, Tomohiro Matsuda, Rob H. A. Verhoeven

**Affiliations:** 1 Department of Research & Development, Netherlands Comprehensive Cancer Organisation (IKNL), Godebaldkwartier 419, 3511 DT Utrecht, The Netherlands; 2 Medical Oncology, Amsterdam UMC Location University of Amsterdam, Amsterdam, The Netherlands; 3 Cancer Center Amsterdam, Cancer Treatment and Quality of Life, Amsterdam, The Netherlands; 4 Department of Surgery, Radboud University Medical Center, Nijmegen, The Netherlands; 5 Department of Head and Neck, Esophageal Medical Oncology, National Cancer Center Hospital, Tokyo, Japan; 6 Department of Medical Oncology, University Medical Center Utrecht, Utrecht University, Utrecht, The Netherlands; 7 Division of International Health Policy Research, Institute for Cancer Control, National Cancer Center Japan, Tokyo, Japan

**Keywords:** Esophageal cancer, Gastric cancer, Metastatic disease, Treatment

## Abstract

**Purpose:**

Differences exist between Asian and Western patients with esophagogastric cancer, for example in terms of histological subtype and treatment strategies. This study aimed to compare characteristics and treatment between patients with metastatic esophagogastric cancer from Japan and the Netherlands using nationwide cancer registry data.

**Methods:**

Patients diagnosed with metastatic esophageal or gastric cancer were included from the nationwide national cancer registry of Japan (2016–2019) and the Netherlands (2015–2020). Treatment strategies were analyzed using chi-squared tests.

**Results:**

The proportion of patients with metastatic esophageal (16.0% vs 34.2%) and gastric cancer (14.9% vs 45.2%) were lower in Japan compared to the Netherlands. Japanese patients with metastatic esophageal adenocarcinoma (EAC), esophageal squamous cell carcinoma (ESCC) or gastric cancer (GC) were more often male and older compared to Dutch patients. Proportion of patients with metastatic disease who received surgical resection was higher in Japan compared to the Netherlands (EAC 9.3 vs 1.4%, *p* < 0.001; ESCC 10.7% vs 2.3%, *p* < 0.001; GC 12.0% vs 3.6% *p* < 0.001). Proportion of patients who received systemic therapy was also higher (EAC 44.8% vs 30.4%, *p* < 0.001; ESCC 26.6% vs 12.0%, *p* < 0.001; GC 50.7% vs 35.8% *p* < 0.001).

**Conclusions:**

Japanese patients less often presented with metastatic esophagogastric cancer and more often underwent surgical resection or received systemic therapy compared to Dutch patients. Further investigation should elucidate what the deliberations are in both Japan and the Netherlands and if more patients in the Netherlands could benefit from surgical resection or systemic therapy and whether this would translate in better survival and quality of life.

**Supplementary Information:**

The online version contains supplementary material available at 10.1007/s00432-023-05111-4.

## Introduction

The incidence of gastric cancer is higher in Japan compared to the Netherlands (global age-standardized incidence rate (ASR) of 31.6 vs 5.2 per 100,000 person-years), while the incidence of esophageal cancer is comparable between the two countries (global ASR of 7.2 vs 6.9 per 100,000 person-years).^(Ferlay J)^ There is however a difference in the prevalence in the main histological subtypes for esophageal cancer between the two countries (Arnold et al. 2020). Esophageal squamous cell carcinoma (ESCC) accounts for approximately 90% of cases in Japan, whereas in the Netherlands for approximately 30% of cases (Saito et al. 2022; van Putten et al. 2018).

In general, palliative systemic therapy is the standard treatment for patients who present with metastatic esophageal or gastric cancer (Lordick et al. 2022; Muro et al. 2019a, b; Obermannova et al. 2022). Global phase III trials for systemic therapy often include patients from Asian and Western countries, with the proportion of Asian patients ranging between 24 and 96%. Davidson and Chau 2016; Kato et al. 2019; Sun et al. 2021; Wilke et al. 2014). A recent meta-analysis including 20 phase II/III trials of patients with unresectable advanced gastroesophageal junction or gastric cancer showed that progression-free survival was comparable between Asian and Western patients, however overall survival was superior in Asian patients (Zhang et al. 2022).

Participation of patients with cancer in clinical trials is limited due to strict criteria (Donnelly et al. 2017) and a comparative study between Asian and Western patients based on unselected groups of patients with metastatic esophagogastric cancer is lacking. A population-based study comparing patients with metastatic esophageal or gastric cancer from Japan and the Netherlands could provide insights in differences and similarities between Asian and Western patients in daily clinical practice. Therefore, this study aimed to compare patient, tumor and treatment characteristics of patients with metastatic esophageal or gastric cancer between Japan and the Netherlands.

## Methods

### Study population

Patients aged 20 years or older diagnosed with esophageal (C15.0-C15.9), gastroesophageal junction/cardia (C16.0) or gastric cancer (C16.1-C16.9) according to ICD-O-3 were selected (Fritz et al. 2000). Data was available from the Japanese Cancer Registry (JCR) of patients diagnosed between 2016–2019 and the Netherlands Cancer Registry (NCR) of patients diagnosed between 2015 and 2020. The JCR serves the total Japanese population and is based on the Cancer Registry Act, which became effective in 2016 and requires all hospitals to submit basic data (including treatment) of newly diagnosed patients to the registry. The information was provided in accordance with the Act (A2020-0018R2) and the data were independently processed by this research team. The NCR serves the total Dutch population and is based on notification of all newly diagnosed malignancies by the national automated pathology archive. Data managers extract information on diagnosis, tumor stage and treatment from medical records for registration in the NCR. Datasets from both countries were merged.

Staging for Dutch patients was recoded according to the Japanese registry as localized, regional lymph nodes involvement, adjacent organ involvement, distant metastases or unknown. Further analyses were only performed for patients with distant metastases and seperataly for esophageal cancer (adenocarcinoma or squamous cell carcinoma) and gastric cancer (including gastroesophageal junction cancer). Tumors were classified as adenocarcinoma, squamous cell carcinoma or carcinoma not otherwise specified (NOS) (Supplementary Table 1) (Fritz et al. 2000). Lauren classification was classified as intestinal, diffuse, mixed, indeterminate, adenocarcinoma NOS or not applicable (Supplementary Table 1). Reason for diagnosis was available for all Japanese patients and for Dutch patients who were diagnosed in 2015.

### Treatment

Treatment was mutually exclusive classified in the following order: surgical resection of the primary tumor (including endoscopic resection), systemic therapy and radiotherapy (directed at the primary tumor; includes concurrent or sequential systemic therapy and radiotherapy), systemic therapy, radiotherapy (directed at the primary tumor), other treatment/best supportive care. Other treatment included argon plasma coagulation, laser therapy, photodynamic therapy, electromagnetic wave coagulation therapy or endocrine treatment (JCR dataset) and metastasectomy or radiotherapy directed at metastases (NCR dataset). Patients with unknown treatment (Japan: *n* = 6298 (6.9%); Netherlands: *n* = 44 (0.5%)) were not included in the analysis according to treatment.

Type of systemic therapy was available for patients who visited the National Cancer Center Hospital (NCCH), Tokyo, Japan between 2018 and 2019 and for all patients diagnosed in the Netherlands. The data were obtained with the consent of the IRB in the NCC and based on the patient’s blanket consent. Type of systemic therapy regimens were classified as previously described (Veer et al. 2016a). In short, regimens were mutually exclusive classified in the following order: trastuzumab containing regimens, non-trastuzumab targeted containing regimens, monotherapy, gemcitabine doublets, cisplatin doublets, fluoropyrimidine doublets, platinum doublets, anthracycline triplets, taxane/irinotecan triplets or unknown regimens.

### Statistical analysis

Characteristics and treatment strategies were displayed with frequencies and percentages and analyzed using chi-squared tests. Two-sided *p*-values of < 0.05 were considered statistically significant. All analyses were conducted using R studio version 4.3.0 and R version 4.1.0.

## Results

**Fig. 1:**
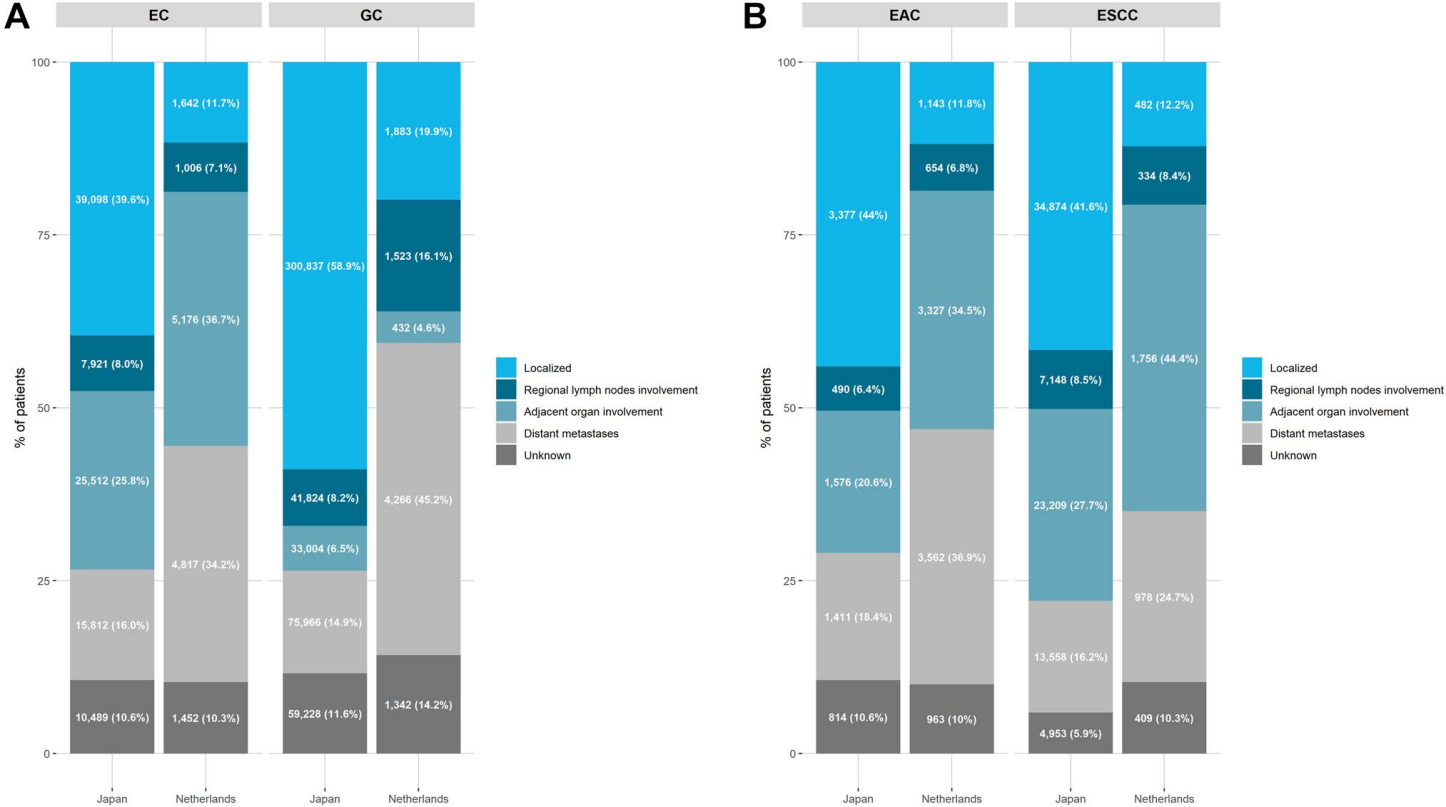
Proportion of patients with esophageal or gastric cancer by stage (**A**) and esophageal adenocarcinoma or esophageal squamous cell carcinoma by stage (**B**). Categories based on clinical TNM classification. Esophageal cancer: localized: T_1-2_N_0_M_0_, regional lymph nodes involvement: T_1-2_N_+_M_0_, adjacent organ involvement: T_3-4_N_all_M_0_ and distant metastases: T_all_N_all_M_1_. Gastric cancer: localized: T_1-3_N_0_M_0_, regional lymph nodes involvement: T_1-3_N_+_M_0_, adjacent organ involvement: T_3-4_N_all_M_0_ and distant metastases: T_all_N_all_M_1_. *EC* esophageal cancer, *GC* gastric cancer, *EAC* esophageal adenocarcinoma, *ESCC* esophageal squamous cell carcinoma

In Japan, among patients diagnosed with esophageal or gastric cancer between 2016 and 2019, 15,812 out of 98,832 (16.0%) and 75,966 out of 510,859 (14.9%) had distant metastatic disease and were included, respectively (Fig. 1). In the Netherlands, among patients diagnosed with esophageal or gastric cancer between 2015 and 2020, 4817 out of 14,093 (34.2%) and 4266 out of 9446 (45.2%) had distant metastatic disease and were included, respectively. Among Japanese patients with metastatic esophageal cancer, 4.9%, 17.8% and 76.8% were diagnosed due to detection in cancer screening or health checks, accidental detection during follow-up of other diseases and due to patient experiencing symptoms, respectively (Supplementary Table 2). Among Japanese patients with metastatic gastric cancer, 5.0%, 21.9% and 72.5% were diagnosed due to detection in cancer screening or health checks, accidental detection during follow-up of other disease and due to patient experiencing symptoms, respectively. Among Dutch patients diagnosed in 2015, 95.3% and 93.3% were diagnosed after experiencing symptoms for metastatic esophageal and gastric cancer, respectively.

### Esophageal cancer

Patients with metastatic esophageal cancer in Japan were older (≥ 80 years: 18.1%) compared to the Netherlands (≥ 80 years: 14.1%; *p* < 0.001) (Table 1). Among patients with metastatic esophageal cancer, Japanese patients more often had a squamous cell carcinoma than Dutch patients (20.3%). For esophageal adenocarcinoma (EAC) (13.9% versus 16.5%; *p* = 0.022) and ESCC (16.1% versus 40.4%; *p* < 001), patients were less often female in Japan compared to the Netherlands (Supplementary Table 3).

**Table 1:** Baseline characteristics for patients with metastatic esophageal or gastric cancer

	Esophageal cancer			Gastric cancer		
	Japan (*N* = 15,812)	Netherlands (*N* = 4817)	*p* value	Japan (*N* = 75,966)	Netherlands (*N* = 4266)	*p* value
Sex			< 0.001			< 0.001
Male	13,265 (83.9%)	3761 (78.1%)		52,037 (68.5%)	2790 (65.4%)	
Female	2547 (16.1%)	1056 (21.9%)		23,929 (31.5%)	1476 (34.6%)	
Age category			< 0.001			< 0.001
< 65	3877 (24.5%)	1676 (34.8%)		13,034 (17.2%)	1378 (32.3%)	
65–79	9078 (57.4%)	2460 (51.1%)		38,774 (51.0%)	2022 (47.4%)	
≥ 80	2857 (18.1%)	681 (14.1%)		24,156 (31.8%)	866 (20.3%)	
Primary tumor location						< 0.001
Esophagus	15,812 (100.0%)	4817 (100.0%)				
Gastroesophageal junction/Cardia				10,192 (13.4%)	1356 (31.8%)	
Gastric				65,774 (86.6%)	2910 (68.2%)	
Histology			< 0.001			0.002
Adenocarcinoma	1411 (8.9%)	3562 (73.9%)		70,371 (92.6%)	4001 (93.8%)	
Squamous cell carcinoma	13,558 (85.7%)	978 (20.3%)		287 (0.4%)	22 (0.5%)	
Carcinoma NOS	843 (5.3%)	277 (5.8%)		5308 (7.0%)	243 (5.7%)	
Lauren classification			< 0.001			< 0.001
Intestinal	319 (2.0%)	1320 (27.4%)		25,933 (34.1%)	1285 (30.1%)	
Diffuse	34 (0.2%)	451 (9.4%)		6783 (8.9%)	1591 (37.3%)	
Mixed	1 (0.0%)	73 (1.5%)		19 (0.0%)	123 (2.9%)	
Interderminate	81 (0.5%)	124 (2.6%)		1016 (1.3%)	77 (1.8%)	
Adenocarcinoma NOS	976 (6.2%)	1594 (33.1%)		36,620 (48.2%)	925 (21.7%)	
Not applicable	14,401 (91.1%)	1255 (26.1%)		5595 (7.4%)	265 (6.2%)	
Tumor differentiation			< 0.001			< 0.001
Well/moderate	4464 (28.2%)	1376 (28.6%)		25,839 (34.0%)	727 (17.0%)	
Poorly/undifferentiated	3039 (19.2%)	1725 (35.8%)		30,366 (40.0%)	1671 (39.2%)	
Unknown	8309 (52.5%)	1716 (35.6%)		19,761 (26.0%)	1868 (43.8%)	

**Fig. 2:**
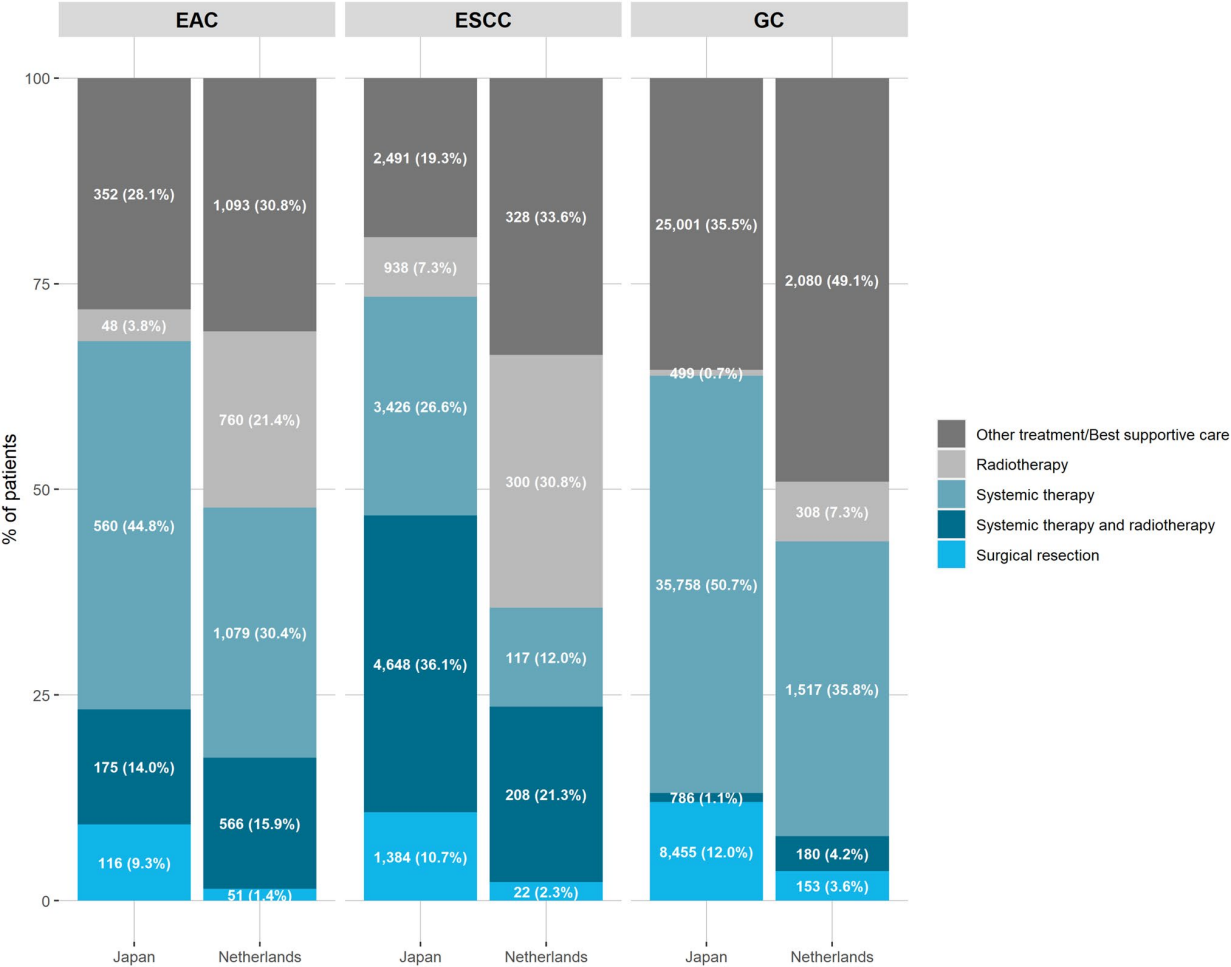
Treatment of patients with metastatic esophageal adenocarcinoma, esophageal squamous cell carcinoma or gastric cancer. Patients with unknown treatment were excluded (Japan: EAC 11.3% (*n* = 160), ESCC 4.9% (*n* = 671), GC 7.2% (*n* = 5467); Netherlands: EAC 0.4% (*n* = 13), ESCC 0.3% (*n* = 3), GC 0.7% (*n* = 28)). *EAC* esophageal adenocarcinoma, *ESCC* esophageal squamous cell carcinoma, *GC* gastric cancer

Among patients with metastatic EAC, in Japan 9.3% underwent surgical resection compared to 1.4% in the Netherlands (*p* < 0.001; Fig. 2). The proportion of patients with metastatic EAC who received systemic therapy was higher in Japan (44.8%) compared to the Netherlands (30.4%, *p* < 0.001), while the proportion of patients who received radiotherapy was lower in Japan (3.8%) compared to the Netherlands (21.4%, *p* < 0.001). The proportion of patients who received other treatment/best supportive care was similar between Japan (28.1%) and the Netherlands (30.8%, *p* = 0.08).

Among patients with metastatic ESCC in Japan, 10.7% underwent surgical resection compared to 2.3% in the Netherlands (*p* < 0.001; Fig. 2). In Japan, the proportion of patients who received systemic therapy and radiotherapy (36.1%) or systemic therapy (26.6%) was higher compared to the Netherlands (systemic therapy and radiotherapy: 21.3%, *p* < 0.001; systemic therapy: 12.0%, *p* < 0.001). In Japan, the proportion of patients who received radiotherapy (7.3%) or other treatment/best supportive care (19.3%) was lower compared to the Netherlands (radiotherapy: 30.8%, *p* < 0.001; other treatment/best supportive care: 33.6%, *p* < 0.001).

Type of systemic therapy was available for 53 and 1,752 patients with metastatic esophageal cancer in Japan and the Netherlands (Supplementary Table 4). Use of cisplatin doublet was higher in Japan (41.5%) compared to the Netherlands (0.6%). The most common regimen in Japan was 5-FU plus cisplatin (35.8%) and in the Netherlands was capecitabine plus oxaliplatin (CapOx; 39.4%).

### Gastric cancer

Among patients with metastatic gastric cancer, the proportion of gastroesophageal junction cancer was lower in Japan (13.4%) compared to the Netherlands (31.88%, *p* < 0.001). Patients with metastatic gastric cancer in Japan were older (≥ 80 years: 31.8%) compared to the Netherlands (≥ 80 years: 20.3%). In Japan less patients had a diffuse type tumor (8.9%) compared to Dutch patients (37.3%).

A higher proportion of patients with metastatic gastric cancer underwent surgical resection in Japan (12.0%) compared to the Netherlands (3.6%, *p* < 0.001). The proportion of patients who received systemic therapy was also higher in Japan (50.7%) compared to the Netherlands (35.8%), *p* < 0.001. Other treatment/best supportive care was less often received by patients in Japan (35.5%) compared to the Netherlands (49.1%, *p* < 0.001).

Type of systemic therapy was available for 133 and 1,679 patients in Japan and the Netherlands, respectively (Supplementary Table 3). The proportion of patients who received a trastuzumab containing regimen was higher in Japan (22.6%) compared to the Netherlands (13.8%), respectively. Only one patient in Japan received triplet therapy (0.8%), while more patients in the Netherlands received triplet therapy (15.6%). The three most common regimens in Japan were S1 plus oxaliplatin (SOX, 23.3%), S1 plus cisplatin (20.3%) and 5-FU plus oxaliplatin (FOLFOX; 15.0%), and in the Netherlands were CapOx (45.0%), FOLFOX (14.4%) and epirubicin, oxaliplatin plus capecitabine (EOX, 8.4%).

## Discussion

Our study showed that the proportion of patients who presented with metastatic disease at diagnosis was lower in Japanese compared to Dutch patients. Additionally, differences exist in characteristics and treatment in patients with metastatic esophageal or gastric cancer between Japan and the Netherlands. Surgical resection rates and systemic therapy administration were higher in Japan compared to the Netherlands.

A lower proportion of female patients was identified for EAC, ESCC and gastric cancer in Japan compared to the Netherlands, particularly striking for EAC (16.1% versus 40.4%). A study investigating global EAC trends reported that in Japan (three regions) and the Netherlands incidence increased for women, while rates for men remained unchanged in Japan and decreased in the Netherlands (Wang et al. 2018). Smoking and alcohol consumption are the two main risk factors for ESCC. According to the Japanese National Health and Nutrition Survey in 2009, smoking rates were 38.2% and 10.9% among men and women, respectively (Ministry of Health, Labour and Welfare. National Health and Nutrition Survey 2009. Available at: http://www.mhlw.go.jp/bunya/kenkou/eiyou/h21-houkoku.html). In the Netherlands data from 2021 stated smoking rates of 24.6% and 16.5% among men and women, respectively (Trimbos instituut. Smoking in the Netherlands: Key statistics for 2021. Available at: https://www.trimbos.nl/aanbod/webwinkel/af1999-smoking-in-the-netherlands-key-statistics-for-2021/). Meaning the proportion of females that smoked compared to men is approximately 1:4 in Japan and approximately 1:2 in the Netherlands, which could explain the lower proportion of females in patients with metastatic EAC in Japan compared to the Netherlands.

A large difference in diffuse type gastric cancer was observed between Japan and the Netherlands. However, in a high proportion of tumors in Japanese patients the Lauren classification was unknown. When comparing the Lauren classification for patients with a known classification, a lower percentage of diffuse type tumors in Japan (20.1%) compared to the Netherlands (51.7%) maintained. This could be explained by the fact that diffuse type gastric adenocarcinoma is more common in the gastroesophageal junction (Koemans et al. 2020) and in our study the proportion of patients with a gastroesophageal junctional tumor was lower in Japan compared to the Netherlands. Additionally, diffuse type gastric cancer is associated with younger age, and the Japanese population was older compared to the Dutch population (Assumpcao et al. 2020; van der Kaaij et al. 2020). Although *Helicobacter pylori* infection is the main risk factor for both the intestinal and diffuse type, small studies in United States (*n* = 59) and in Japan (*n* = 68) reported a higher prevalence of *Helicobacter pylori* in intestinal type as opposed to diffuse type gastric cancer (Endo et al. 1995; Parsonnet et al. 1991). According to a global meta-analysis the estimated prevalence of *Helicobacter pylori* infections is higher in Japan (51.7%) compared to the Netherlands (35.5%) (Hooi et al. 2017). Prognosis of patients with intestinal or diffuse type tumor differs, patients with an intestinal type have a better prognosis compared to patients with a diffuse type (Petrelli et al. 2017). Due to the higher percentage of tumors with an unknown Lauren classification these results should be interpreted with caution.

The proportion of patients who received surgical resection or systemic therapy was higher in Japan than the Netherlands. The extent of metastases could have been limited in Japanese compared to Dutch patients due to cancer screening or during follow-up for other diseases resulting in a lower tumor burden and higher possibility of tumor-directed treatment options. Additionally, in Japan esophageal cancer metastases in the supraclavicular lymph nodes are considered regional as opposed to a distant (which were registered as M1 in the JCR according to the TNM classification). Therefore these patients were eligible for surgical resection and could explain the higher resection rate (Japan Esophageal Society 2017). In gastric cancer, if Japanese patients present with resectable liver metastases or metastases limited to the para-aortic lymph node resection of the primary tumor is still weakly recommended (Japanese Gastric Cancer Association 2022). A recent population-based study in the Netherlands showed that resection of the primary tumor in patients with metastases limited to the liver diagnosed between 2015 and 2017 was limited to 1% (Kroese et al. 2022).

Results in our study regarding type of systemic therapy in Japanese patients could be unrepresentative for the total Japanese population as type of regimen was only available in one Japanese hospital. Although, in our study the most common regimens among Japanese patients with metastatic gastric cancer were SOX and S1 plus cisplatin in line with previous publications (Komatsu et al. 2022; Takashima et al. 2009; Yamada 2020). Among Dutch patients with metastatic gastric cancer the most common regimen was CapOx. Capecitabine and S-1 are similar types of oral fluoropyrimidine drugs, but most evidence for S-1 has been established in Asian patients and none of the Dutch patients in our study received S-1 (Jin et al. 2008; Xu et al. 2013). A recent meta-analysis reported that S-1 based regimens are effective and tolerable in first-line for advanced gastric cancer in both Asian and Western countries (Ter Veer et al. 2016b) Hand-foot syndrome, a potential side effect of chemotherapy, was found to be lower in patients receiving S-1 as compared to capecitabine. Most likely due to differences in metabolism of S-1 between Asian and Western patients, dose tolerability is lower in Western patients (Ma et al. 2010).

In EAC, 5-FU plus cisplatin is the standard regimen used in the Japanese clinical practice and was also most commonly administrated in our study (Hiramoto et al. 2018; Japan Esophageal Society 2017). Dutch guidelines recommend the use of oxaliplatin instead of cisplatin due to favorable toxicity profile and outpatient treatment (“Dutch Clinical Practice Guidelines for Gastric Carcinoma—Eerstelijns systemische behandeling,”). As opposed to gastric cancer, for which a Japanese study found similar effectiveness and a favorably toxicity profile for SOX compared to S1 plus cisplatin therapy (Yamada et al. 2015), a direct comparison of oxaliplatin versus cisplatin doublets in EAC is lacking. As a result, 5-FU plus cisplatin remains the standard of care in Japan.

The strength of our study is the use of population-based data, which represents all patients diagnosed in daily clinical practice in both countries. Our study has several limitations. In the JCR information on comorbidities, performance status, location and number of metastatic sites was unavailable, which could have resulted in an even better comparison between the two countries. Additionally, it was unknown if Japanese patients received chemotherapy with concurrent or sequential radiotherapy, therefore a distinction between chemoradiotherapy or palliative radiotherapy for symptoms could not be performed. Due to difference in coding of topography, comparison of primary tumor location within the esophagus or stomach was not possible. For both the JCR and NCR accurate information on reason(s) for not giving a certain type of treatment was unavailable and therefore it is difficult to determine why there are differences in treatment between both countries. Finally, information on survival was unavailable in the present study due to logistics constrains.

In conclusion, our study observed a higher proportion of Japanese patients with metastatic esophageal or gastric cancer who underwent surgical resection or received systemic therapy compared to Dutch patients. Whether this were patients with oligometastatic disease/low metastatic burden is unknown. Further research should elucidate if more patients in the Netherlands could benefit from surgical resection or systemic therapy and whether this would translate in improved survival and better quality of life.

### Supplementary Information

Below is the link to the electronic supplementary material.

Supplementary file1 (DOCX 41 KB)

## Data Availability

The data underlying this article is available at the Netherlands Comprehensive Cancer Organisation (IKNL) and at the Japanese Ministry of Health, Labour and Welfare through the National Cancer Center upon justified request.
