# Supplementary material for: A population-based comparison of patients with metastatic esophagogastric carcinoma between Japan and the Netherlands
Source: J Cancer Res Clin Oncol. 2023 Jul 24;149(14):13323–30. doi: 10.1007/s00432-023-05111-4 (PMC10587097; doi:10.1007/s00432-023-05111-4)
Supplement: Supplementary file 1 — Supplementary file1 (DOCX 41 KB) [file 432_2023_5111_MOESM1_ESM.docx]

**Supplementary table 1. Classification of histology and Lauren classification according to the third edition of the International Classification of Diseases for Oncology.**

| **Classification** | **Subgroup** | **Morphology codes** |
| --- | --- | --- |
| **Histology** | Adenocarcinoma | 8140, 8142-8145, 8190, 8201, 8210, 8211, 8230, 8255, 8260-8263, 8310, 8323, 8401, 8471, 8480, 8481, 8490, 8510, 8512, 8530, 8560, 8570, 8571, 8572, 8574, 8576 |
|  | Squamous cell carcinoma | 8050-8084 |
| **Lauren classification^1^** | Intestinal | 8144, 8201, 8211, 8260, 8262 |
|  | Diffuse | 8142, 8145, 8490 |
|  | Mixed | 8255 |
|  | Indeterminate | 8480, 8481, 8510, 8512, 8560, 8570 |
|  | Adenocarcinoma NOS | 8140, 8143, 8190, 8210, 8230, 8261, 8263, 8310, 8323, 8401, 8471, 8530, 8571, 8572, 8574, 8576 |

NOS: not otherwise specified.

^1^Squamous cell carcinoma and carcinoma NOS were classified as ‘not applicable’.

**Supplementary table 2. Reason for diagnosis for patients with metastatic esophageal or gastric cancer.** Reasons for diagnosis was available for all patients in Japan and for patients diagnosed in 2015 in the Netherlands.

|  | **Esophageal cancer** | | | **Gastric cancer** | | |
| --- | --- | --- | --- | --- | --- | --- |
|  | **Japan**  (N=15,812) | **Netherlands**  (N=787) | **p value** | **Japan**  (N=75,966) | **Netherlands**  (N=668) | **p value** |
| **Reason for diagnosis** |  |  | <0.001 |  |  | <0.001 |
| Accidental detection during follow-up of other diseases | 2821 (17.8%) | 21 (2.7%) |  | 16605 (21.9%) | 20 (3.0%) |  |
| Detection in cancer screening or health checks | 778 (4.9%) | NA^1^ |  | 3776 (5.0%) | NA^1^ |  |
| Due to patient experiencing symptoms | 12143 (76.8%) | 750 (95.3%) |  | 55104 (72.5%) | 623 (93.3%) |  |
| Other or unknown reason | 70 (0.4%) | 16 (2.0%) |  | 481 (0.6%) | 25 (3.7%) |  |

^1^For the general Dutch population surveillance or screening for esophageal or gastric cancer does not occur in the Netherlands.

**Supplementary table 3. Baseline characteristics of patients with metastatic esophageal adenocarcinoma or squamous cell carcinoma.**

|  | **Adenocarcinoma** | | | **Squamous cell carcinoma** | | |
| --- | --- | --- | --- | --- | --- | --- |
|  | **Japan** (N=1411) | **Netherlands** (N=3562) | **p value** | **Japan** (N=13558) | **Netherlands** (N=978) | **p value** |
| **Sex** |  |  | 0.022 |  |  | <0.001 |
| Male | 1215 (86.1%) | 2974 (83.5%) |  | 11374 (83.9%) | 583 (59.6%) |  |
| Female | 196 (13.9%) | 588 (16.5%) |  | 2184 (16.1%) | 395 (40.4%) |  |
| **Age category** |  |  | <0.001 |  |  | <0.001 |
| <65 | 490 (34.7%) | 1300 (36.5%) |  | 3258 (24.0%) | 306 (31.3%) |  |
| 65-80 | 649 (46.0%) | 1790 (50.3%) |  | 8029 (59.2%) | 546 (55.8%) |  |
| ≥80 | 272 (19.3%) | 472 (13.3%) |  | 2271 (16.8%) | 126 (12.9%) |  |
| **Lauren** **classification** |  |  | <0.001 |  |  |  |
| Intestinal | 319 (22.6%) | 1320 (37.1%) |  |  |  |  |
| Diffuse | 34 (2.4%) | 451 (12.7%) |  |  |  |  |
| Mixed | 1 (0.1%) | 73 (2.0%) |  |  |  |  |
| Interderminate | 81 (5.7%) | 124 (3.5%) |  |  |  |  |
| Adenocarcinoma NOS | 976 (69.2%) | 1594 (44.8%) |  |  |  |  |
| Not applicable |  |  |  | 13558 (100.0%) | 978 (100.0%) |  |
| **Tumor** **differentiation** |  |  | <0.001 |  |  | <0.001 |
| Well/moderate | 498 (35.3%) | 1019 (28.6%) |  | 3965 (29.2%) | 356 (36.4%) |  |
| Poorly/undifferentiated | 439 (31.1%) | 1326 (37.2%) |  | 2555 (18.8%) | 309 (31.6%) |  |
| Unknown | 474 (33.6%) | 1217 (34.2%) |  | 7038 (51.9%) | 313 (32.0%) |  |

**Supplementary table 4. Type of systemic therapy regimen.** Patients who received surgical resection and/or chemoradiotherapy were excluded.

|  | **Esophageal cancer** | | | **Gastric cancer** | | |
| --- | --- | --- | --- | --- | --- | --- |
|  | **Japan** (N=53) | **Netherlands** (N=1752) | **p value^1^** | **Japan** (N=133) | **Netherlands** (N=1679) | **p value^1^** |
| **Main drug classification** |  |  | <0.001 |  |  | <0.001 |
| Monotherapy | 1 (1.9%) | 62 (3.5%) |  | 12 (9.0%) | 115 (6.8%) |  |
| F-doublet (FP, FT, FI) | 3 (5.7%) | 898 (51.3%) |  | 52 (39.1%) | 1008 (60.0%) |  |
| C-doublet (CF, CT, CE) | 22 (41.5%) | 11 (0.6%) |  | 27 (20.3%) | 5 (0.3%) |  |
| G-doublet (GP, GC) | 0 (0.0%) | 7 (0.4%) |  | 0 (0.0%) | 6 (0.4%) |  |
| P-doublet (PT, PE) | 0 (0.0%) | 276 (15.8%) |  | 0 (0.0%) | 29 (1.7%) |  |
| A-triplet (ACF, AFOx) | 0 (0.0%) | 98 (5.6%) |  | 0 (0.0%) | 186 (11.1%) |  |
| T- or I-triplet (TCF, FOxT, IFOx) | 9 (17.0%) | 37 (2.1%) |  | 1 (0.8%) | 76 (4.5%) |  |
| Trastuzumab containing regimen | 7 (13.2%) | 341 (19.5%) |  | 30 (22.6%) | 231 (13.8%) |  |
| Non-trastuzumab targeted containing regimen^2^ | 11 (20.8%) | 12 (0.7%) |  | 11 (8.3%) | 13 (0.8%) |  |
| Unknown | 0 (0.0%) | 10 (0.6%) |  | 0 (0.0%) | 10 (0.6%) |  |
| **Specific drug classification**^3^ |  |  | <0.001 |  |  | <0.001 |
| S1 | 1 (1.9%) | 0 (0.0%) |  | 10 (7.5%) | 0 (0.0%) |  |
| Capecitabine and oxaliplatin (CapOx) | 0 (0.0%) | 690 (39.4%) |  | 0 (0.0%) | 756 (45.0%) |  |
| S1 and oxaliplatin (SOX) | 2 (3.8%) | 0 (0.0%) |  | 31 (23.3%) | 0 (0.0%) |  |
| S1 and cisplatin | 3 (5.7%) | 0 (0.0%) |  | 27 (20.3%) | 0 (0.0%) |  |
| 5-FU and oxaliplatin (FOLFOX) | 1 (1.9%) | 190 (10.8%) |  | 20 (15.0%) | 241 (14.4%) |  |
| 5-FU and cisplatin | 19 (35.8%) | 2 (0.1%) |  | 0 (0.0%) | 0 (0.0%) |  |
| Carboplatin and paclitaxel | 0 (0.0%) | 270 (15.4%) |  | 0 (0.0%) | 27 (1.6%) |  |
| Epirubicin, oxaliplatin and capecitabine (EOX) | 0 (0.0%) | 87 (5.0%) |  | 0 (0.0%) | 141 (8.4%) |  |
| 5-FU, cisplatin and docetaxel (DCF) | 9 (17.0%) | 1 (0.1%) |  | 1 (0.8%) | 4 (0.2%) |  |
| Capecitabine, oxaliplatin and trastuzumab | 0 (0.0%) | 200 (11.4%) |  | 1 (0.8%) | 116 (6.9%) |  |
| Capecitabine, cisplatin and trastuzumab | 0 (0.0%) | 60 (3.4%) |  | 9 (6.8%) | 44 (2.6%) |  |
| S1, oxaliplatin and trastuzumab | 2 (3.8%) | 0 (0.0%) |  | 12 (9.0%) | 0 (0.0%) |  |
| Other | 16 (30.2%) | 252 (14.4%) |  | 22 (16.5%) | 350 (20.8%) |  |

^1^Fisher exact test

^2^Eight Japanese patients received non-trastuzumab targeted containing regimen or placebo due to enrollment in a clinical trial.

^3^If occurring in at least 5% of patients in either the Japanese or Dutch population.

A: anthracycline, C: cisplatin, E: etoposide, F: fluoropyrimidine, G: gemcitabine; I: irinotecan, Ox: oxaliplatin, P: platinum, T: taxane.
